# Impact of California’s Senate Bill 27 on Antimicrobial-Resistant Escherichia coli Urinary Tract Infection in Humans: Protocol for a Study of Methods and Baseline Data

**DOI:** 10.2196/45109

**Published:** 2023-05-05

**Authors:** Ana Florea, Joan A Casey, Keeve Nachman, Lance B Price, Magdalena E Pomichowski, Harpreet S Takhar, Vanessa Quinlivan, Lee D Childs, Meghan F Davis, Rong Wei, Vennis Hong, Jennifer H Ku, Cindy M Liu, Alice Pressman, Sarah Robinson, Katia J Bruxvoort, S Bianca Salas, Sara Y Tartof

**Affiliations:** 1 Department of Research & Evaluation Kaiser Permanente Southern California Pasadena, CA United States; 2 Columbia University Mailman School of Public Health New York City, NY United States; 3 Johns Hopkins Bloomberg School of Public Health Baltimore, MD United States; 4 Milken Institute School of Public Health George Washington University Washington, DC United States; 5 Johns Hopkins School of Medicine Baltimore, MD United States; 6 Center for Health Systems Research Sutter Health Walnut Creek, CA United States; 7 Department of Epidemiology University of Alabama at Birmingham Birmingham, AL United States; 8 Department of Health Systems Science Kaiser Permanente Bernard J Tyson School of Medicine Pasadena, CA United States

**Keywords:** AMR, antimicrobial resistance, E coli, Escherichia coli, urinary tract infection, UTI

## Abstract

**Background:**

Overuse of antibiotics contributes to antimicrobial resistance (AMR) and is a growing threat to human health worldwide. Previous work suggests a link between antimicrobial use in poultry and human AMR extraintestinal pathogenic *Escherichia coli* (E coli) urinary tract infections (UTIs). However, few US-based studies exist, and none have comprehensively assessed both foodborne and environmental pathways using advanced molecular and spatial epidemiologic methods in a quasi-experimental design. Recently, California enacted Senate Bill 27 (SB27), which changed previous policy to require a veterinarian’s prescription for the use of antibiotic drugs, and which banned antibiotic use for disease prevention in livestock. This provided an opportunity to evaluate whether SB27 will result in a reduction in antimicrobial-resistant infections in humans.

**Objective:**

We describe in detail the methods implemented to achieve the overarching objective of this study to evaluate the impact of SB27 on downstream antibiotic resistance rates in human UTIs.

**Methods:**

A summary of the overall approach and the partnerships between Columbia University, George Washington University (GWU), Johns Hopkins Bloomberg School of Public Health, Kaiser Permanente Southern California (KPSC) Research and Evaluation, the Natural Resources Defense Council, Sanger Institute at Stanford University, Sutter Health Center for Health Systems Research, the University of Cambridge, and the University of Oxford is presented. The collection, quality control testing, and shipment of retail meat and clinical samples are described. Retail meat (chicken, beef, turkey, and pork) was purchased from stores throughout Southern California from 2017 to 2021. After processing at KPSC, it was shipped to GWU for testing. From 2016 to 2021, after clinical specimens were processed for routine clinical purposes and immediately before discarding, those with isolated colonies of *E coli*, *Campylobacter*, and *Salmonella* from KPSC members were collected and processed to be shipped for testing at GWU. Detailed methods of the isolation and testing as well as the whole-genome sequencing of the meat and clinical samples at GWU are described. KPSC electronic health record data were used to track UTI cases and AMR patterns among the cultured specimens. Similarly, Sutter Health electronic health record data were used to track UTI cases in its Northern California patient population.

**Results:**

From 2017 to 2021, overall, 12,616 retail meat samples were purchased from 472 unique stores across Southern California. In addition, 31,643 positive clinical cultures were collected from KPSC members during the same study period.

**Conclusions:**

Here, we presented data collection methods for the study, which was conducted to evaluate the impact of SB27 on downstream antibiotic resistance rates in human UTI. To date, it is one of the largest studies of its kind to be conducted. The data collected during this study will be used as the foundation for future analyses specific to the various objectives of this large body of work.

**International Registered Report Identifier (IRRID):**

DERR1-10.2196/45109

## Introduction

Worldwide, antibiotics are used to prevent and treat bacterial infections in both humans and animals. However, routine and inappropriate uses of antibiotics have contributed to antimicrobial resistance (AMR), which is a major challenge to human health worldwide [1]. In the United States, industrial food animal production drives antibiotic consumption through both therapeutic and prevention uses in animals [2]. The majority of medically important antibiotics are sold for use in livestock, with tetracyclines accounting for 66% of these antibiotics [3,4]. This livestock use may result in antimicrobial-resistant infections in humans. Prior work [5,6] has suggested a link between antibiotic use in poultry and antimicrobial-resistant extraintestinal pathogenic *Escherichia coli (E coli)* urinary tract infections (UTIs) in humans [7-11]. This suggests the potential for use of antibiotics in food-producing animals to be important to address to help curb AMR.

California has a large livestock industry and is one of the leading US producers of beef cattle, broiler chickens, and turkey [12,13]. It is also the most populous US state, with a human population larger than the Netherlands, Denmark, and Sweden combined (countries that lead the European Union science and policy on AMR in livestock [14,15]). In an effort to reduce AMR and related human infections, on January 1, 2018, California enacted Senate Bill 27 (SB27), which changed previous policy to require a veterinarian’s prescription for the use of antibiotic drugs, and banned antibiotic uses for disease prevention in livestock [16]. A similar policy was enacted in the European Union in 2006, which led to reductions in antibiotic use and AMR [14,17-20]. SB27 was the first such legislation in the United States and provided a natural experiment with which to investigate whether a ban on antimicrobials used for disease prevention in livestock was associated with a decrease in antimicrobial-resistant *E coli* in retail meat and among UTI cases in humans.

Here, we describe the methods of a study that was a collaborative effort among Columbia University, George Washington University (GWU), Johns Hopkins Bloomberg School of Public Health (JHU), Kaiser Permanente Southern California (KPSC) Research and Evaluation, the Natural Resources Defense Council, Sanger Institute at Stanford University, Sutter Health Center for Health Systems Research, University of Cambridge, and the University of Oxford. Specifically, we describe the methods used to investigate whether the passage and implementation of SB27 were associated with a decrease in AMR among retail meat products in California and among *E coli* UTI at KPSC and Sutter. We simultaneously collected chicken, beef, turkey, and pork retail meat samples from across Southern California and clinical isolates from KPSC between 2017 and 2021. We also leveraged KPSC electronic health record (EHR) data to track UTI cases and AMR patterns among the cultured specimens. Similarly, Sutter Health EHR data were used to track UTI cases in its Northern California patient population. Whole-genome sequencing (WGS) was also performed by GWU on confirmed *E coli*, *Salmonella*, and *Campylobacter* isolates from retail meat and clinical samples.

Taken together, the aims of the study were to characterize and compare AMR phenotypes, genes, and *E coli* populations isolated from retail chicken and human clinical samples; to quantify changes in AMR phenotypes, genes, and *E coli* populations from retail chicken and human clinical samples before and after SB27 implementation; and to define the spatial relationship between livestock operations and patients with an antimicrobial-resistant *E coli* UTI. Under SB27, California was the first US state to ban nontherapeutic uses of antibiotics in livestock. At the time of this writing, this was the first population-based study in the United States to rigorously measure the association between AMR profiles of *E coli* isolated from chicken retail meat and human clinical samples during the implementation of a statewide policy meant to reduce antibiotic use. This descriptive assessment builds on a small number of early publications [16,21-24] to provide a common foundation for future analyses specific to the various objectives of this large body of work.

## Methods

### Partnerships and Summary of Approach

#### Meat and Human Clinical AMR Data

A prospective study design was used over the study period (2016-2021) to collect information on AMR in retail meat and human infections. Retail meat was purchased in Southern California and human infection data came from KPSC in Southern California and Sutter Health in Northern California. Each week, retail meat (chicken, turkey, beef, and pork) was purchased from stores across Southern California. Clinical and demographic data for members presenting with a UTI were extracted from the KPSC EHR along with ancillary information on *E coli* isolated from urine specimens. Study data were also obtained for KPSC members with *Salmonella* and *Campylobacter* isolated from stool samples. Each week all isolated *Salmonella* and *Campylobacter* and a subset of the isolated *E coli*, based on convenience sampling (samples processed across multiple shifts from Bacteriology), were collected from KPSC laboratories for additional analysis. Similarly, in Northern California, Sutter Health’s EHR data were used to extract clinical and demographic data for patients who presented with a UTI; however, no clinical samples were collected from Sutter Health. The purchased meat and the subset of clinical isolates from KPSC were shipped to GWU (Price Laboratory). GWU isolated bacteria from the meat and conducted antimicrobial susceptibility testing of *E coli, Salmonella*, and *Campylobacter* from meat (Table S2 in Multimedia Appendix 1). Additionally, human isolates from KPSC were sent to GWU for further susceptibility testing to augment EHR resistance profiles with microbials that were not tested by the KPSC clinical laboratory.

#### Additional Partnerships

The study involved several additional key partnerships to conduct laboratory analyses, supply supplemental clinical data, identify livestock operation locations, and describe SB27 implementation. The Sanger Institute, the University of Oxford, and the University of Cambridge partnered with GWU on the WGS data management, genome assembly, quality control analysis, and sequence type assignment of select samples. The RegLab at Stanford University [25] developed a methodology to map animal production sites using satellite imagery and will be utilized in future spatial epidemiologic analyses to assess whether proximity to animal production sites increases the risk for AMR UTI; Beckton, Dickinson, and Company provided national data on UTI for a manuscript on UTI pre- and post-SB27; and KPSC used a local laboratory in Pasadena (EMSL Analytical, Inc [EMSL]) for quality control of meat testing. Finally, JHU partnered with the study team to (1) conduct parallel analyses of microbial recovery and AMR from the National AMR Monitoring System within and beyond California [26], and (2) undertake a qualitative study of food-animal producers and related agricultural stakeholders within and beyond California to describe attitudes, knowledge, beliefs, and practices around antimicrobial uses in food animal production, including in the context of SB27 [21].

**Figure 1:**
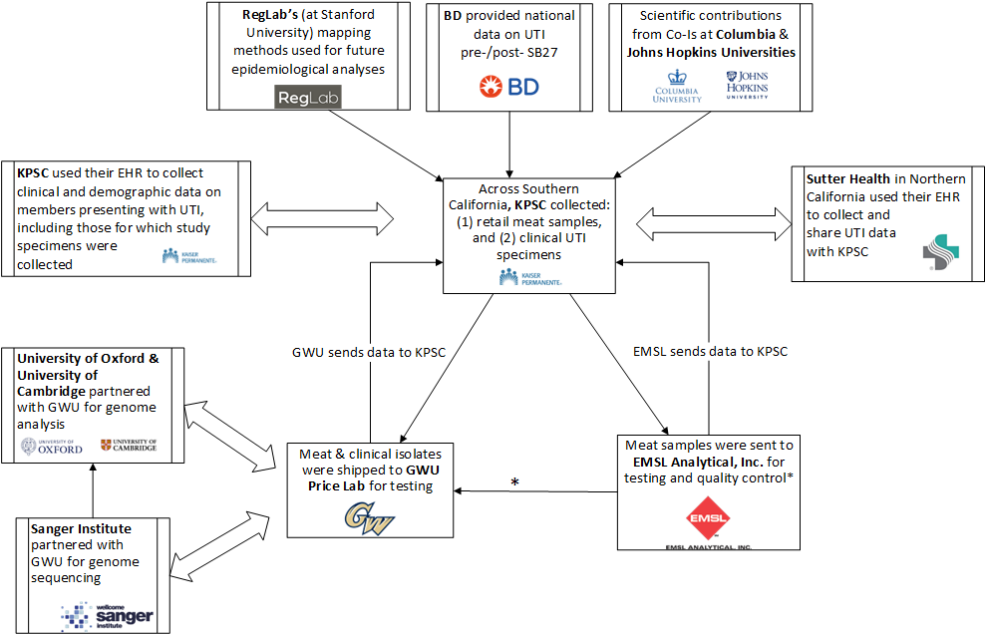
Study partnerships flowchart. Meat collection paused due to the pandemic. Once we resumed meat collection during lockdown, samples were sent to EMSL only as GWU was closed. Once GWU re-opened, meat isolates were shipped from EMSL to GWU. BD: Beckton, Dickinson, and Company; EHR: electronic health record; GWU: George Washington University; KPSC: Kaiser Permanente Southern California; SB27: Senate Bill 27; UTI: urinary tract infection.

**Figure 2:**
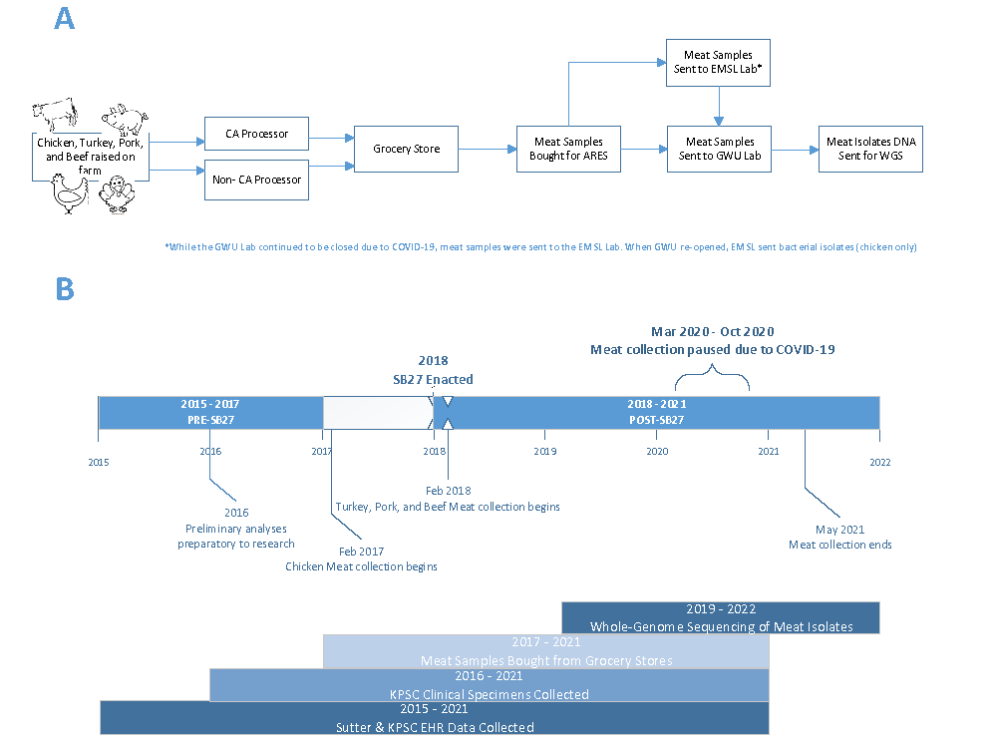
Summarized study activities and timelines. (A) Flowchart of meat samples from farm to sequencing. (B) Timelines for all study components. ARES: Antimicrobial resistant Escherichia coli before and after California Senate Bill 27; CA: California; EHR: electronic health record; EMSL: EMSL Analytical, Inc.; GWU: George Washington University; KPSC: Kaiser Permanente Southern California; SB27: Senate Bill 27; WGS: whole-genome sequencing.

The partnerships are summarized in Figure 1. Timelines for EHR data, meat collection, and clinical sample collection activities are summarized in Figure 2.

### Retail Meat Samples

#### Collection and Shipment

Meat sample collection occurred from 2017 to 2021 (Figure 2B) in regions where KPSC facilities were located (from Bakersfield to San Diego). To ensure that the retail meat-sampling approach maximized the diversity of geography and meat processor facilities, from March through July 2017, we performed several inventory surveys of hundreds of stores to collect data on availability and attributes (eg, regulated label claims like United States Department of Agriculture [USDA]–certified organic or raised without antibiotics [if neither, then classified as “no restrictions”], and if processed in California or non-California) of meat. Research associates (RAs) surveyed stores across Southern California (Los Angeles, Inland Empire, and San Diego areas), taking photos of retail meat package labels and gathering all USDA Establishment (meat processor) code. Establishment codes are used to identify the facility in which the retail meats are processed [27]. These codes include p-codes used for poultry (chicken and turkey) and m-codes used for pork and beef. Surveys were repeated yearly to ensure ongoing representation of USDA establishment codes available for purchase in the retail establishments in our target region. The survey data informed the development of weekly shopping routes for retail meat collection, each of which included a list of designated stores and meat types to purchase. There were 53 different routes, each with assigned stores. The Los Angeles region was divided into 8 sections with 42 routes, the Inland Empire region had 6 routes, and the San Diego region had 5 routes. A Google Maps (Multimedia Appendix 2) account was used to document and save the routes.

The RAs followed prespecified criteria for retail meat purchasing. They were instructed to purchase smaller packages, valued under US $10 and weighing less than 2 lbs, when possible. Larger packages were permitted when no smaller packages existed for the brand or establishment code combination of interest. Only raw meat that had not undergone any secondary processing was purchased. Expiration or sell-by dates were to be at least 3 days after the date of purchase and the longest expiration period was to be purchased when possible. The RAs traveled with labels, cooler, ice packs, temperature gauge, assigned shopping list, Ziploc bags, paper towels, gloves, and insulated shopping bags. They also used a prepaid debit card to monitor the study budget.

After meat was purchased, a study ID was assigned. The shopping date was written on the ID label and placed close to the printed price label. Two pictures of the meat with the label (one of the whole package and the other, a close-up of the establishment code and label) were taken. A third photo was taken if the establishment code was not easily visible in the close-up photo. Sealed retail meat packages were then placed into a Ziploc bag with a paper towel. Retail meat packages were transported to the KPSC research facility, the same day of purchase, in a cooler, equipped with a thermometer, where the meat was kept cool, but never frozen; temperature logs were also kept.

Following transport to the research facility, retail meat packages were placed in a study refrigerator. Photos were uploaded by the RA into the secure study folder and saved per instructions in the standard operating procedures (SOPs). All meat-related data and photos were also entered into the study tracking database; a list of the variables in the tracking database is provided in Multimedia Appendix 3. This database was only accessible to approved study staff and saved in a secure KPSC network study folder. Tracking ensured the correct amounts of retail meat were purchased, shipments were sent on time every Tuesday to be received by GWU no later than Friday, and for other general tracking needs. The study tracking database was created using Microsoft Access and maintained by a data manager. The data manager was also responsible for all troubleshooting and for updating the database as needed. The database stored data on meat samples, tracked clinical samples, and shipping data.

Each meat sample was individually placed in a second Ziploc bag with a paper towel to prevent any leaks or cross-contamination. The paper towel was placed on the backside of the meat package to maintain the visibility of the ID labels. The individually packaged, double-bagged samples were placed into a study cooler. Ice packs wrapped in insulation were placed on the bottom of the cooler, followed by a double layer of meat samples. This was repeated until the cooler was full (approximately 3-4 rows of meat samples per cooler), and ice packs were added as the top layer. The temperature was monitored in all the study coolers.

Each cooler had a “Research Purposes Only” label attached on the outside, and a KPSC business card on the inside. The cooler was securely sealed before shipment through FedEx overnight “First Priority” to the GWU laboratory. If a FedEx pick-up could not be scheduled from the KPSC research facility, the designated RA would drop off the shipment at the nearest FedEx location. All shipment related details were tracked in the study tracking database.

During meat purchasing, transferring, and shipping, Occupational Safety and Health Administration (OSHA) standards and guidelines were followed by all study staff. All RAs were cross-trained in meat collection, tracking, safety, and shipping. RA meetings were used to help address or resolve study issues or concerns. Participants in these meetings included the study team, project coordinator or project manager (PM), and GWU (as needed).

#### Quality Control Testing

KPSC used EMSL, a laboratory located in Pasadena, which performs bacterial testing of meat, for quality control of the study retail meat samples. Each week, approximately 5-10 samples were randomly provided to EMSL for quality control. EMSL used their own SOPs adapted to align with SOPs developed by GWU.

#### The COVID-19 Pandemic

In March 2020, the study was paused through the middle of October 2020 due to the COVID-19 pandemic as staff was required to work from home. After the study resumed, EMSL became the primary laboratory as the GWU laboratory continued to be closed to in-person work. During April-May 2021, the GWU laboratory resumed on-site work and all retail meat isolates, including any samples tested by EMSL during the closure were sent to GWU for processing and testing.

### Clinical Samples

#### Collection and Shipment

After clinical specimens were processed for routine clinical purposes and immediately before discarding, clinical specimens in the form of agar plates with isolation colonies of *E coli, Campylobacter,* and *Salmonella* from KPSC members were collected from Regional KPSC labs on a weekly basis from 2016 to 2021.

Under aseptic conditions, *E coli* was isolated by KPSC study laboratory technicians from 100 mm Blood Agar- Trypticase soy agar II MacConkey/ChomAGAR Biplates into Biobank tubes (Microbank tubes with beads, Pro-Lab Diagnostics) under a vented CLASS II hood. *Campylobacter* was isolated from *Campylobacter* blood agar plates and placed into Biobank tubes. *Salmonella* was isolated from Trypticase soy agar slants and placed into Biobank tubes. All Biobank tubes were labeled with custom −80 °C-resistant study labels and were stored frozen at −80 °C before shipping. All samples were maintained in the freezer and temperature logs were checked weekly to ensure no temperature deviations occurred. Samples were shipped through FedEx Priority Overnight First Delivery to GWU for testing. Before shipping samples to GWU, a GPS temperature monitoring probe was used to confirm the temperatures were acceptable for transport. In addition, through our study database, we were able to accurately track all samples and their location.

All clinical sample data were tracked in the study database that also maintained the retail meat sample data. Laboratory RAs entered the patient medical record number, sample type, accession number, study ID, and other variables outlined in Multimedia Appendix 4. Study IDs were used to maintain the privacy and security of the protected health information of the patient and their sample. This database was also used to generate on-demand reports of the number of samples collected and the demographic characteristics of the patients from whom the clinical samples were collected.

### Testing of Meat and Clinical Samples at GWU

#### Isolation and Testing

To isolate bacterial species of interest from meat, 200±25 g of each retail meat specimen was aseptically transferred to a stomacher bag and 250±25 g of the appropriate enrichment broth was added. The bags were placed on a stomacher rack, briefly agitated on a benchtop shaker, then placed in an incubator. Species-specific selective and screening media were inoculated from the enrichment broth cultures, and several rounds of single-colony isolation were performed to obtain pure isolates of *E coli*, *Salmonella*, and *Campylobacter*. The species of each isolate was confirmed by quantitative polymerase chain reaction. *E coli* was isolated from all 4 meat types; *Salmonella* was isolated from chicken, turkey, and pork; and *Campylobacter* was isolated from chicken. Species-specific enrichment and isolation methods for each bacterial species are further detailed in Multimedia Appendix 1.

The “Antibiotic Susceptibility Testing” section in Multimedia Appendix 1 describes both types of tests conducted at GWU (Kirby-Bauer and Minimum Inhibitory Concentration [MIC]) to test *E coli, Salmonella* spp, and *Campylobacter* spp isolates for susceptibility to a panel of antibiotics. The Kirby-Bauer disc test was conducted on all meat isolates, as well as for the subset of human clinical isolates. MIC tests were conducted on a subset of both meat and human clinical isolates to augment K-B disc testing. Specifically, MIC testing by the broth microdilution method was conducted for antibiotics to which >5% of *E coli* or *Salmonella* isolates from any source were resistant.

Most of the susceptibility data for *E coli* from clinical isolates came from testing conducted as part of routine clinical microbiology protocols at KPSC; MIC data were obtained with the Beckton, Dickinson, and Company Phoenix method. During GWU’s COVID-19 closure, when EMSL conducted testing of some meat samples, similar procedures and protocols were followed.

#### Preparation of Samples for WGS and Sequencing Procedures

DNA for genomic analysis was extracted from a subsample of the confirmed meat and clinical sample-derived *E coli*, *Salmonella* spp, and *Campylobacter* spp isolates using the PureLink Pro 96 Genomic DNA Purification Kit (Invitrogen). WGS was performed on the NovaSeq platform with a read length of 150 bp. Assemblies for downstream analyses were generated from raw sequencing data, and quality control of the sequencing run was performed by checking the quality of the trimmed sequencing reads. Multilocus sequence typing was performed for *E coli* using the Achtman scheme [28,29] and population structure was inferred using fastbaps. A midpoint-rooted species tree was built under a GTR+GAMMA substitution model. Additional detail on methods for WGS data processing and analysis is available in Multimedia Appendix 1.

### EHR Data

KPSC and Sutter Health integrated health care systems have wide coverage areas, high completeness of data, racial, ethnic, and socioeconomic diversity among their members or patients, longitudinal health care usage information, and research infrastructure [30,31]. Both systems use Epic for their EHR data, allowing the synthesis of information across the 2 systems. Data were aggregated across both networks to identify UTI cases presenting for clinical care across California populations, thereby increasing the representation and generalizability of the results.

KPSC provides comprehensive care to more than 4.7 million members across 15 medical centers and 235 medical offices in Southern California. KPSC uses KP HealthConnect (an Epic system) as its EHR to capture all aspects of members’ clinical care, encounters and communications, and administrative and demographic data. HealthConnect links all patient care information including primary and specialist care, outpatient surgery, laboratory services, radiology, pharmacy, patient-level demographic, and membership data and billing. The system links facilities across the region and provides members, physicians, and other authorized health care providers with online access to clinical information.

In Northern California, Sutter Health, a not-for-profit organization, provides care to more than 3 million patients across 22 counties, including rural, urban, and suburban communities. Like KPSC, Sutter Health uses a comprehensive Epic EHR system allowing researchers to access detailed clinical data, including physician orders and free text clinical notes, as well as the standard ambulatory, hospital, laboratory, and pharmacy data.

Researchers from KPSC and Sutter Health partnered to develop reproducible programing code to construct standardized data sets from the EHR representing episodes of UTI from 2015 to 2022. This collaboration included monthly meetings to discuss data extractions, share code, and monitor data quality. Early publications on trends in UTI and risk factors for UTI, including low socioeconomic status and ambient temperature, have been conducted on these data sets [22-24].

All analyses were conducted using SAS Enterprise Guide 8.2 (SAS Institute) at KPSC, and R at Sutter Health.

### Training

All study staff were trained on study procedures by the PM. The PM had OSHA certification and trained staff on how to safely handle meat samples during the collection and shipment. SOPs were drafted to ensure consistency and accuracy in specimen collection, and a Project Management Plan was developed to ensure that all study milestones were met on time.

### Ethics Approval

No patient recruitment or patient contact occurred as part of this study, and all study data will be limited [32]. Both health systems use an opt-out research participation system where members or patients can opt out of their health data being used for research. If they do not opt out, their data become available for research following institutional review board (IRB) approval and Health Insurance Portability and Accountability Act (HIPAA) compliance.

The study was approved by the Kaiser Permanente Southern California (KPSC) IRB (IRB #11284); GWU and JHU ceded review to the KPSC IRB. A waiver for the use of protected health information and of informed consent was approved for this study by the KPSC IRB. The study was also approved by the Sutter Health IRB (IRBNet #1063246) with a waiver of HIPAA authorization and informed consent. No IRB was required from the remaining collaborating sites as no data were collected at those sites.

## Results

### Retail Meat Samples

Overall, retail meat samples were purchased from 472 unique stores. Between 2017 and 2021, chicken retail meat samples were purchased from 285 unique stores (Table 1). Starting in 2018, the study initiated the purchase of retail turkey, pork, and beef meat samples from 266, 267, and 253 stores, respectively (Table 1). Multimedia Appendix 2 shows the map of the stores throughout the Southern California region.

A total of 12,616 retail meat samples were collected from the 472 stores from 2017 to 2021 (Table 2). Of these, 6084 were chicken, 2701 were turkey, 2486 were beef, and 1345 were pork.

**Table 1:** Unique stores visited for retail chicken, turkey, pork, and beef meat purchases (2017-2021).

	Total number of unique stores visited, n						Total^a^
	2017	2018	2019	2020	2021		
Chicken	96	244	256	135	75	285	
Turkey	N/A^b^	244	240	121	53	266	
Pork	N/A	245	249	121	50	267	
Beef	N/A	216	233	118	45	253	

^a^Total of unique stores visited.

^b^N/A: not applicable.

**Table 2:** Meat samples (chicken, turkey, pork, and beef) collected (2017-2021; N=12,616).

Type of meat	Meat samples, n^a^ (%)^b^					
	2017	2018	2019	2020	2021	Total
Chicken	1180 (19)	1964 (32)	2212 (36)	420 (7)	308 (5)	6084
Turkey	N/A^c^	979 (36)	1324 (49)	279 (10)	119 (4)	2701
Pork	N/A	442 (33)	719 (53)	133 (10)	51 (4)	1345
Beef	N/A	896 (36)	1259 (51)	233 (9)	98 (4)	2486

^a^n: sample size.

^b^Row percentages.

^c^N/A: not applicable.

Table 3 further details the retail chicken, turkey, pork, and beef samples collected from 2017 to 2021 by the state of processing (California and non-California) and by regulated label (USDA-certified organic, raised without antibiotics, and no restrictions). Among the retail chicken, pork, and beef samples bought, more came from a California processor than a non-California one. For the chicken, 1529 USDA-certified organic samples were identified. For the beef, 605 samples were identified as organic. Furthermore, for the pork, none of the samples were organic. Conversely, for turkey, non-California samples were more prevalent, with 441 being organic.

**Table 3:** Meat samples (chicken, turkey, pork, and beef) collected by year, state of processing, and regulated label (2017-2021). All meat isolates were purchased in California.

			Meat samples, n^a^																		
			Total^b^							Processed in California^b,c^							Processed outside of California^b,c^				
			Organic		RWA^d^		No restrictions		Organic			RWA^d^		No restrictions		Organic			RWA^d^		No restrictions
**Chicken**																					
	Total^e^	1529		3959		2041		1208			3113		1660		316			822		292	
	2017	247		584		530		209			490		468		37			91		45	
	2018	483		1319		639		381			1015		497		101			297		107	
	2019	599		1519		684		467			1188		541		129			319		115	
	2020	117		303		115		87			232		93		30			71		17	
	2021	83		234		73		64			188		61		19			44		8	
**Turkey**																					
	Total^f^	441		927		1771		383			529		531		58			396		1238	
	2018	133		340		638		131			203		182		2			136		455	
	2019	235		452		871		197			252		263		38			199		607	
	2020	48		92		187		40			54		64		8			38		123	
	2021	25		43		75		15			20		22		10			23		53	
**Pork**																					
	Total^g^	0		129		1216		0			106		366		0			8		142	
	2018	0		36		406		0			30		132		0			4		51	
	2019	0		68		651		0			56		184		0			4		75	
	2020	0		17		116		0			15		33		0			0		11	
	2021	0		8		43		0			5		17		0			0		5	
**Beef**																					
	Total^h^	605		1236		1228		418			776		807		186			452		391	
	2018	200		433		456		124			249		264		75			177		170	
	2019	326		626		619		229			404		434		97			222		177	
	2020	54		119		113		43			80		80		11			39		33	
	2021	25		58		40		22			43		29		3			14		11	

^a^n: sample size.

^b^Categories “Organic,” “RWA,” and “No Restrictions” are not mutually exclusive.

^c^Establishment codes were used to assign California versus non-California processing location; samples missing these codes were thus not assigned to either group.

^d^RWA: raised without antibiotics.

^e^Total: n=6084, processed in California: n=4847, and processed outside of California: n=1124 (for the latter 2, missing establishment codes (n): Chicken: 113).

^f^Total: n=2701, processed in California: n=1062, and processed outside of California: n=1635 (for the latter 2, missing establishment codes (n): Turkey: 4).

^g^Total: n=1345, processed in California: n=472, and processed outside of California: n=150 (for the latter 2, missing establishment codes (n): Pork: 723).

^h^Total: n=2486, processed in California: n=1589, and processed outside of California: n=859 (for the latter 2, missing establishment codes (n): Beef: 38).

### Clinical Samples

A total of 31,643 positive clinical cultures were collected from KPSC members from 2017 through 2021 (Table 4). Of these, 28,647 tested positive for *E coli*, 1681 for *Campylobacter*, and 1315 for *Salmonella.*

Overall, the mean age of KPSC members with a positive clinical specimen was 49.3 (SD 23.1) years (Table 5). This group was also primarily female (85.7%, 27,104/31,643) and Hispanic (44.1%, 13,945/31,643).

**Table 4:** Clinical specimens of Kaiser Permanente Southern California (KPSC) members collected (2017-2021; N=31,643).

Bacterium type		Clinical specimens, n^a^ (%)^b^					
		2017	2018	2019	2020	2021	Total
	*Escherichia coli*	4351 (15)	8285 (29)	8492 (30)	6269 (22)	1250 (4)	28,647
	*Campylobacter*	254 (15)	479 (28)	623 (37)	271 (16)	54 (3)	1681
	*Salmonella*	214 (16)	417 (32)	392 (30)	236 (18)	56 (4)	1315

^a^n: sample size.

^b^Row percentages.

**Table 5:** Demographic characteristics of Kaiser Permanente Southern California (KPSC) members with positive clinical specimens (2017-2021).

			*Escherichia coli* (N=28,647)		*Campylobacter* (N=1681)		*Salmonella* (N=1315)		Total (N=31,643)
Age (years), mean (SD)			50.9 (22.5)		36.4 (22.1)		31 (24.9)		49.3 (23.1)
**Sex, n^a^ (%)**									
	Female	25,632 (89.5)		785 (46.7)		687 (52.2)		27,104 (85.7)	
	Male	3015 (10.5)		896 (53.3)		628 (47.8)		4539 (14.3)	
**Race or ethnicity^b^, n (%)**									
	Hispanic	12,391 (43.3)		870 (51.8)		684 (52)		13,945 (44.1)	
	White	10,864 (37.9)		503 (29.9)		412 (31.3)		11,779 (37.2)	
	Asian	2292 (8)		175 (10.4)		106 (8.1)		2573 (8.1)	
	Black	1894 (6.6)		40 (2.4)		54 (4.1)		1988 (6.3)	
	Pacific Islander	168 (0.6)		19 (1.1)		6 (0.5)		193 (0.6)	
	Native Am Alaskan	76 (0.3)		2 (0.1)		2 (0.2)		80 (0.3)	
	Am Indian or Alaska native	2 (0)		0 (0)		0 (0)		2 (0)	
	Other	344 (1.2)		25 (1.5)		19 (1.4)		388 (1.2)	
	Multiple	114 (0.4)		8 (0.5)		6 (0.5)		128 (0.4)	
	Unknown	488 (1.7)		36 (2.1)		26 (2)		550 (1.7)	
	Decline to state	13 (0)		3 (0.2)		0 (0)		16 (0.1)	

^a^n: sample size.

^b^Missing: n=1 for *E coli*.

## Discussion

We present data collection methods for the study which was conducted to help fill a gap in the literature by evaluating the impact of SB27 on downstream antibiotic resistance rates in human UTIs. To date, it is one of the largest studies of its kind to evaluate the relationship between antibiotic use in livestock production and AMR profiles in retail meat and humans before and after the implementation of a statewide policy meant to reduce antibiotic use in the livestock sector.

The study concurrently sampled retail meat (chicken, turkey, beef, and pork) and clinical specimens at regular time intervals. The study team cultured and isolated *E coli*, *Salmonella*, and *Campylobacter* from retail meat, and clinical specimens were processed by clinical laboratory staff at KPSC to isolate bacteria of interest. WGS was performed on a subset of *E coli* isolates, and all *Salmonella* and *Campylobacter* isolates. This study used expansive EHR data, before (2015-2017) and after (2018-2021) SB27 enactment, from KPSC and Sutter Health, 2 large and well-established health systems. Overall, 12,616 samples of meat were collected from more than 400 retail stores in Southern California. From 31,643 clinical specimens, 28,647 *E coli*, 1315 *Salmonella*, and 1681 *Campylobacter* isolates were collected. Our large study sample size thus allows us to support emerging evidence of the connection between AMR in livestock and human *E coli* UTI [33,34]. This study had complementary methodological components requiring different measurement techniques and areas of expertise, including laboratory-based methods, geospatial modeling, market-basket research design and techniques, incorporation of publicly available data sets, and use of secondary EHR data for population health research. Moving forward, the study data will be used for further analysis and manuscripts that address the project’s various primary and secondary aims, as well as complementary work and secondary data analyses through various partnerships.

The study had strengths and limitations. It successfully collected retail meat, clinical microbiological specimens, and data over a 5-year period. Early in the study, there were no readily available data to help identify Concentrated Animal Feeding Operation locations. As a result, we sought out and partnered with Stanford University to classify Concentrated Animal Feeding Operation locations. To set up the study, huge, concerted logistical planning across various divisions had to be implemented. The coordination of laboratory leadership at KPSC to integrate research into the clinical flow had to occur, as well as hiring a large RA team to cover the large Southern California area needed to collect the meat samples. The RAs also had to ensure all the meat samples bought were kept at reasonable temperatures from purchase to delivery to GWU where they could properly be tested. The shipments themselves were an undertaking as they tended to be bulky and heavy, and they needed their temperatures constantly monitored. Additionally, we worked with the EMSL laboratory to mirror the main laboratory (GWU) procedures, to act as a quality control laboratory for the meat samples. Though the RA team covered many grocery stores throughout Southern California, we did not collect data on which products were more likely to be purchased by KPSC members. However, we strategically selected a representative sample of grocery stores to overlap with the geographic distribution of KPSC facilities and members. The study was also successful in gaining access to the clinical samples, and in being able to collect a large quantity for the study. A further limitation was the lack of routine testing for tetracycline for clinical UTI samples at KPSC. As a solution, we shipped all study samples to GWU for tetracycline susceptibility testing. Lastly, the COVID-19 pandemic impacted data collection, as testing laboratories were closed for several months, and meat purchasing was suspended for a short period due to safety concerns of field staff. However, data collection resumed in mid-October 2020 with additional pandemic procedures in place, such as the addition of personal protective equipment and other safety precautions.

This methods paper describes the processes of collecting, processing, and shipping meat and clinical samples, and can be used as a guide for any subsequent, similar research. The analyses implemented and subsequent results will provide the evidence necessary to evaluate the effectiveness of SB27 and will be detailed in forthcoming publications.

## Appendix

Multimedia Appendix 1 GWU Isolation and Testing Methods.

Multimedia Appendix 2 Store distribution for collection of meat samples.

Multimedia Appendix 3 Data collected for each meat sample purchased.

Multimedia Appendix 4 Data collected for each clinical sample.

## Abbreviations

AMR: antimicrobial resistance
EHR: electronic health record
EMSL: EMSL Analytical, Inc
GWU: George Washington University
HIPAA: Health Insurance Portability and Accountability Act
IRB: institutional review board
JHU: Johns Hopkins Bloomberg School of Public Health
KPSC: Kaiser Permanente Southern California
MIC: minimum inhibitory concentration
OSHA: Occupational Safety and Health Administration
PM: project manager
RA: research associate
SB27: Senate Bill 27
SOP: standard operating procedure
USDA: United States Department of Agriculture
UTI: urinary tract infection
WGS: whole-genome sequencing

## Data Availability

Anonymized data that support the findings of this study may be made available from the investigative team in the following conditions: (1) agreement to collaborate with the study team on all publications, (2) provision of external funding for administrative and investigator time necessary for this collaboration, (3) demonstration that the external investigative team is qualified and has documented evidence of training for human subjects protections, and (4) agreement to abide by the terms outlined in data use agreements between institutions.
