# Supplementary material for: Impact of California’s Senate Bill 27 on Antimicrobial-Resistant Escherichia coli Urinary Tract Infection in Humans: Protocol for a Study of Methods and Baseline Data
Source: JMIR Res Protoc. 2023 May 5;12:e45109. doi: 10.2196/45109 (PMC10199382; doi:10.2196/45109)
Supplement: Multimedia Appendix 1 [file resprot_v12i1e45109_app1.docx]

## **APPENDIX 1**. GWU Isolation and Testing Methods

**Isolation of *E. coli*, *Salmonella spp.*, and *Campylobacter spp.* from retail meat specimens**

**Enrichment of meat specimens for bacterial species of interest**

To isolate bacterial species of interest, 200 + 25g of each retail meat specimen was aseptically transferred to a stomacher bag and 250 + 25g of the appropriate enrichment broth was added. The bags were placed on a stomacher rack, briefly agitated on a benchtop shaker, then placed in an incubator. *E. coli* was isolated from all four meat types; *Salmonella* was isolated from chicken, turkey, and pork; and *Campylobacter* was isolated from chicken. Species-specific enrichment and isolation methods are summarized in **Table S1**.

**Table S1.**

| **Species** | **Enrichment conditions** | | **Isolation media** | |
| --- | --- | --- | --- | --- |
|  |  |  | **Stage 1** | **Stage 2 + 3** |
| *E. coli* | MacConkey Broth  16-24 hours at 42^o^C | | VRBA | UTI Chromogenic agar |
| *Salmonella* | Day 1:  Buffered Peptone Water:  16-24 hours at 37^o^C | Day 2:  RVS:  16-24 hours at 42^o^C  MKTTN:  16-24 hours at 37^o^C | XLD agar | *Salmonella* Chromogenic agar |
| *Campylobacter* | Bolton Broth  4 hours at 37^o^C, then 36-44 hours at 42^o^C | | Cefex | mCCDA |

mCCDA: modified Charcoal Cefoperazone Deoycholate Agar; MKTTN: Mueller-Kauffman Tetrathionate Novobiocin broth; RVS: Rappaport Vassiliadis Soya broth; UTI: Urinary Tract Infection; VRBA: Violet Red Bile Agar; XLD: Xylose Lysine Deoxycholate

***E. coli* isolation**

MacConkey broth (prepared from dehydrated media, VWR) selects for gram negative bacteria including *E. coli.* Following incubation, a glycerol stock was prepared from each enrichment culture. A 10uL aliquot of each enrichment culture was then streaked onto a Violet Red Bile Agar with MUG (VRBA) plate (prepared from dehydrated media, VWR) and incubated for 2 hours at 37^o^C, then overnight at 42^o^C (**Table 1**). VRBA selects for gram-negative bacteria and allows color-based screening for lactose fermentation. The four colonies that best resembled an *E. coli* control (ATCC 25922) were selected from each VRBA plate and streaked on a HardyCHROM UTI chromogenic differential agar plate (Hardy Diagnostics) divided into quadrants, then incubated overnight at 37^o^C. After a second round of isolation on chromogenic agar, one isolated colony from each specimen was selected and streaked onto Luria-Bertani (LB; prepared from dehydrated media, VWR) agar. Species confirmation was performed by qPCR with a hydrolysis probe targeting the *uidA* gene (forward primer: 5′-CGTATCACNGTTTGTGTGAACAA-3′, reverse primer: 5’-GGATTCACNACTTGCAAAGTC-3’, probe: 5’-VIC-AACTGGCAGACTATCC- NFQMGB-3’). Isolates confirmed as *E. coli* were preserved as glycerol stocks and subjected to antibiotic susceptibility testing.

***Salmonella* isolation**

To continue enrichment for *Salmonella spp.*, two aliquots were taken from each culture in non-selective Buffered Peptone Water (prepared from dehydrated media, VWR) and transferred to culture tubes containing Rappaport Vassiliadis Soya Broth (RVS; prepared from dehydrated media, VWR) and Mueller-Kauffman Tetrathionate Novobiocin Broth (MKTTN; prepared from dehydrated media, MKTTN novobiocin supplement, and iodine-iodide solution; Sigma-Aldrich) respectively. RVS and MKTTN are selective media for enrichment of *Salmonella*. After a second overnight incubation, glycerol stocks were prepared from each enrichment culture. A 10uL aliquot of each enrichment culture was then streaked onto a Xylose Lysine Deoxycholate (XLD; prepared from dehydrated media, VWR) agar plate and incubated overnight at 37^o^C. XLD contains sodium deoxycholate to inhibit growth of gram-positive bacteria and contains dyes that produce a color change with exposure to hydrogen sulfide produced by *Salmonella* as it metabolizes lysine. The four colonies derived from each specimen that best resembled a *Salmonella* control (ATCC 13076) were selected and streaked onto HardyCHROM *Salmonella* differential chromogenic agar (Hardy Diagnostics), then incubated overnight at 37^o^C. After a second round of isolation on chromogenic agar, one isolated colony from each specimen was selected and streaked onto LB agar. Isolates were confirmed as *Salmonella* by qPCR with a hydrolysis probe targeting the *invA* gene (forward primer: 5’-AGCGTACTGAAAGGGAAAG-3’, reverse primer: 5’- ATACCGCCAATAAAGTTCACAAAG-3’, probe: 5’- HEX-CGTCACCTT /ZEN/ TGATAAACTTCATCGCA-IBFQ-3’). Positively identified *Salmonella* isolates were preserved as glycerol stocks and subjected to antibiotic susceptibility testing.

***Campylobacter* isolation**

Following incubation in Bolton broth (prepared from dehydrated media and Bolton selective supplement, VWR, and supplemented with 5% horse blood, Lampire), a glycerol stock was prepared from each enrichment culture. Bolton broth contains ingredients that minimize damage from oxygen exposure, and several antibiotics used to select for *Campylobacter spp.*  A 10uL aliquot of each enrichment culture was then streaked onto Campy-Cefex agar (VWR) and incubated 48-72h in microaerophilic conditions at 42^o^C. Cefex contains cefoperazone to select for *Campylobacter spp.* The four colonies that best resembled a *C. jejuni* control strain (ATCC 33560) were then streaked onto a modified Charcoal Cefoperazone Deoycholate Agar (mCCDA, VWR) plate and incubated 48-72h in anaerobic conditions at 42^o^C. mCCDA is a selective medium for *Campylobacter spp.* After a second round of isolation on mCCDA, one isolated colony from each specimen was selected and streaked onto Mueller-Hinton agar with 5% sheep blood (VWR) and maintained in anaerobic conditions. Isolates were confirmed as *Campylobacter* by a hydrolysis probe-based qPCR assay targeting a sequence within the 16s rRNA gene conserved among *Campylobacter* species (forward primer: 5’- CGTGCTACAATGGCATATACAATGA -3’, reverse primer: 5’- CGATTCCGGCTTCATGCTC-3’, probe: 5’-VIC-CAGAGAACAATCCGAACTG-NFQMGB-3’) (adapted from Lund et al, 2004; PMID: 15528705). Positively identified *Campylobacter* isolates were preserved as glycerol stocks and subjected to antibiotic susceptibility testing.

**Whole-Genome Sequencing**

Genomic DNA was extracted from a subsample of the confirmed *E. coli*, *Salmonella spp.*, and *Campylobacter spp.* isolates using the PureLink Pro 96 Genomic DNA Purification Kit (Invitrogen). Whole-genome sequencing was performed on the NovaSeq platform with a read length of 150 bp.

WGS data was processed and analyzed using the tools listed below, which may be downloaded from the following conda channels:

*Channels*

- conda-forge

- bioconda

- r

*Dependencies*

- fastqc=0.11.9

- adapterremoval=2.3.2

- spades=3.15.3

- mlst=2.19.0

- r-fastbaps=1.0.4

- panaroo=1.2.8

- iqtree=2.2.0_beta

- ariba=2.14.6

- abricate=1.0.1

- snakemake-wrapper-utils=0.2.0

- snakemake-minimal=6.2.0

First the raw reads have their sequencing adapters removed with adapterremoval, excluding any reads less than 15 bp long and excluding any singletons. The fastqc program is run to check the quality of the sequencing and spot any outliers. The spades program is then run with the --careful flag activated, and the resulting scaffold output fasta files are used for downstream analyses. MLST typing is performed with the mlst software, and fastbaps is used for lineage clustering. The clustering is not constrained by a phylogeny, and instead the baps model is specified. The assembled scaffolds are further annotated by prokka, and the resulting gff files are analyzed by panaroo for the calculation of the pangenome. The core genome alignment is then extracted and used to build a midpoint rooted species tree with iqtree2 , under a GTR+GAMMA substitution model.

**Antibiotic Susceptibility Testing**

*E. coli*, *Salmonella spp.*, and *Campylobacter spp.* isolates were tested for susceptibility to a panel of antibiotics by disc diffusion (CLSI M02: Performance Standards for Antimicrobial Disk Susceptibility Tests, 13^th^ edition). A small amount of each isolate was suspended in PBS by vortexing and adjusted to turbidity equivalent to a 0.5 McFarland standard. Within 15 minutes, each *E. coli* or *Salmonella* suspension was spread evenly over the surface of a Mueller-Hinton Agar (MHA, VWR) plate using a sterile cotton swab. The inoculated plates were allowed to absorb excess moisture for 3-5 minutes at room temperature, then paper discs containing the antibiotics to be tested were tamped onto the surface of the plate using a dispenser. Plates were incubated inverted at 37^o^C for 16-24 hours. For *Campylobacter*, MHA + 5% blood (VWR) was used, and plates were incubated in anaerobic conditions at 42^o^C for 24 hours. Zones of inhibition (ZOI) were measured to the nearest millimeter and were used to categorize each isolate as susceptible or resistant to each antibiotic according to published CLSI breakpoints (**Table S2**).

**Table S2.**

| **Antibiotic** | **Amount on Disc** | **Organism(s) Tested^1^** | **ZOI Ranges (mm)** | | |
| --- | --- | --- | --- | --- | --- |
|  |  |  | **Susceptible** | **Intermediate** | **Resistant** |
| Amoxicillin-Clavulanic Acid | 20/10 ug | E, S | >= 18 | 14-17 | <= 13 |
| Ampicillin | 10 ug | E, S | >= 17 | 14-16 | <= 13 |
| Azithromycin^2^ | 15 ug | E, S | >= 13 | -- | <= 12 |
| Cefazolin | 30 ug | E | >= 15 | -- | <= 14 |
| Cefoxitin | 30 ug | E, S | >= 18 | 15-17 | <= 14 |
| Ceftiofur | 30 ug | E, S | >= 21 | 18-20 | <= 17 |
| Ceftriaxone | 30 ug | E | >= 23 | 20-22 | <= 19 |
|  |  | S | >= 21 | 14-20 | <= 13 |
| Chloramphenicol | 30 ug | E, S | >= 18 | 13-17 | <= 12 |
| Ciprofloxacin | 5 ug | E | >= 26 | 20-25 | <= 21 |
|  |  | C | >= 24 | 21-23 | <= 20 |
|  |  | S | >=31 | 21-30 | <=20 |
| Doxycycline | 30 ug | E | >= 14 | 11-13 | <= 10 |
| Ertapenem | 10 ug | E | >= 22 | 19-21 | <= 18 |
| Erythromycin | 15 ug | C | >= 16 | 13-15 | <=12 |
| Gentamicin | 10 ug | E, S | >= 15 | 13-14 | <= 12 |
| Imipenem | 10 ug | E | >= 23 | 20-22 | <= 19 |
| Kanamycin | 30 ug | E | >= 18 | 14-17 | <= 13 |
|  |  | S | >= 16 | 14-15 | <= 13 |
| Meropenem | 10 ug | E | >= 23 | 20-22 | <= 19 |
| Nalidixic Acid^3^ | 30 ug | E, S | >= 19 | 14-18 | <= 13 |
| Streptomycin^4^ | 10 ug | E, S | >= 15 | 12-14 | <= 11 |
| Sulfisoxazole | 250 ug | E, S | >= 17 | 13-16 | <= 12 |
| Tetracycline | 30 ug | E | >= 15 | 12-14 | <= 11 |
|  |  | S | >= 19 | 15-18 | <= 14 |
|  |  | C | >= 26 | 23-25 | <= 22 |
| Trimethoprim-Sulfamethoxazole | 1.25 ug | E, S | >= 16 | 11-15 | <= 10 |

^1^E = *E. coli*, S = *Salmonella spp.*, C = *Campylobacter spp.*

^2^*E. coli* breakpoints are not available, so the *Salmonella* breakpoints were applied for both organisms.

^3^ Salmonella breakpoints are not available, so the *E. coli* breakpoints were applied for both organisms.

^4^Breakpoints were assigned based on the BD BBL Sensi-Disc product literature.

Minimum Inhibitory Concentration (MIC) testing by the broth microdilution method was performed for antibiotics to which >5% of *E. coli* or *Salmonella* isolates from any source were resistant. Concentration ranges to be tested were selected based on the range of MICs reported in the 2018 NARMS Retail Meat Report. Antibiotic panels of 2-fold serial dilutions were prepared in 96-well plates and inoculated with isolates to be tested according to a protocol adapted from *CLSI Publication M07: Methods for Dilution Antimicrobial Susceptibility Tests for Bacteria That Grow Aerobically*. After 16-20 hours incubation, the optical density of each well was measured at 620 nm using a plate reader. Wells with OD620 > 0.1 were considered positive for bacterial growth. MICs were recorded and the susceptibility of each isolate to each antibiotic was determined based on the CLSI breakpoints and NARMS interpretive standards summarized in **Table S3**.

**Table S3.**

| **Antibiotic** | **Organism(s) Tested^1^** | **Range Tested (ug/mL)** | **MIC Ranges (ug/mL)** | | |
| --- | --- | --- | --- | --- | --- |
|  |  |  | **Susceptible** | **Intermediate** | **Resistant** |
| Amoxicillin-Clavulanic Acid | E, S | 0.5/0.25-64/32 | <= 8/4 | 16/8 | >= 32/16 |
| Ampicillin | E, S | 0.5-64 | <= 8 | 16 | >= 32 |
| Azithromycin^2^ | E, S | 0.25-16 | <= 16 | n/a | >= 32 |
| Cefazolin | E, S | 1-64 | <= 2 | 4 | >= 8 |
| Cefoxitin | S | 0.004-16 | <= 8 | 16 | >= 32 |
| Ceftriaxone | E, S | 2-64 | <= 1 | 2 | >= 4 |
| Chloramphenicol | S | 0.5-64 | <= 8 | 16 | >= 32 |
| Doxycycline | E | 0.25-64 | <= 4 | 8 | >= 16 |
| Gentamicin | E | 2-256 | <= 4 | 8 | >= 16 |
| Kanamycin | E | 2-128 | <= 16 | 32 | >= 64 |
| Streptomycin^2^ | E, S | 4-256 | <=16 | n/a | >= 32 |
| Sulfisoxazole | E, S | 8-512 | <= 256 | n/a | >= 512 |
| Tetracycline | E, S | 2-64 | <= 4 | 8 | >= 16 |
| Trimethoprim-Sulfamethoxazole | E, S | 0.002/0.037 – 0.063/1.19 | <= 2/38 | n/a | >= 4/76 |

^1^E = *E. coli*, S = *Salmonella spp*.

^2^Breakpoints were assigned based on the interpretive standards used in the 2018 NARMS Retail Meat Report.
