# Supplementary material for: Impact of California’s Senate Bill 27 on Antimicrobial-Resistant Escherichia coli Urinary Tract Infection in Humans: Protocol for a Study of Methods and Baseline Data
Source: JMIR Res Protoc. 2023 May 5;12:e45109. doi: 10.2196/45109 (PMC10199382; doi:10.2196/45109)

## APPENDIX 2: Store distribution for collection of meat samples

**Los Angeles Route**


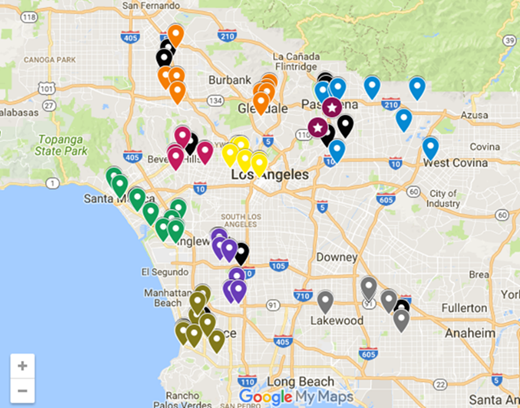


**Inland Empire & San Diego Route**


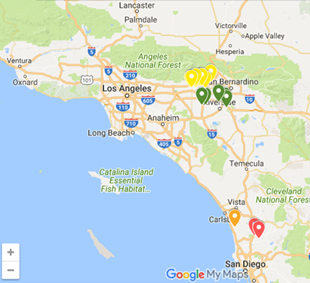

Supplement: Multimedia Appendix 2 [file resprot_v12i1e45109_app2.docx]
