# Supplementary material for: Impact of California’s Senate Bill 27 on Antimicrobial-Resistant Escherichia coli Urinary Tract Infection in Humans: Protocol for a Study of Methods and Baseline Data
Source: JMIR Res Protoc. 2023 May 5;12:e45109. doi: 10.2196/45109 (PMC10199382; doi:10.2196/45109)
Supplement: Multimedia Appendix 3 [file resprot_v12i1e45109_app3.docx]

## **APPENDIX 3**. Data collected for each meat sample purchased

- **Grocery Store Name/Location**
  - Grocery Store Name
  - Address
  - City
  - State
  - Zip
  - Initials of RA
  - Region
- **Meat Description**
  - Study ID (the next available number)
  - Meat Brand
  - P-code
  - Meat Cut (if applicable)
  - Net Weight
  - Price
  - Visit Date
  - Expire Date
  - Purchased Indicator (always true)
  - No ABX, Organic and Value Pack check boxes (optional)
  - (Note: Some chicken was not "purchased" by our Research Associates and does not have a study number. Documented for research/prep purposes)
- **EMSL Lab Drop Off** (completed only if dropped off at EMSL)
  - Drop Off Date
  - Lab Result
  - Drop Off Temperature
- **Shipped Meat** (completed if shipped to George Washington University/John Hopkins University)
  - Shipped Date
  - Current Sample Location
  - Shipped To
  - Fed Ex Tracking Number
- **Chicken Picture**
  - JPEG Photo of the Chicken Package
