# Supplementary material for: Impact of California’s Senate Bill 27 on Antimicrobial-Resistant Escherichia coli Urinary Tract Infection in Humans: Protocol for a Study of Methods and Baseline Data
Source: JMIR Res Protoc. 2023 May 5;12:e45109. doi: 10.2196/45109 (PMC10199382; doi:10.2196/45109)
Supplement: Multimedia Appendix 4 [file resprot_v12i1e45109_app4.docx]

## **APPENDIX 4**. Data collected for each clinical sample

- **Isolate Record**
  - Study ID
  - MRN
  - ARES Box Number
  - Position Number
  - Accession Number
  - Organism Type
  - In-Patient (Y/N)
  - Test Code
  - Bacti Date
  - Biobank Tube Date
  - Sample Origin
  - Funding Type
  - Result
  - Associate Name
- **Shipment Information**
  - Current Sample Location
  - Shipped To
  - Shipped Date
  - Fed Ex Tracking Number
  - Test Result/Comment
